# Books and Authors

**Published:** 1910-08

**Authors:** 


### BOOKS AND AUTHORS

**Duodenal Ulcer.** By **B. G. A. Moynihan,** M.S. (London) F.R.C.S., Senior Assistant Surgeon at Leeds General Infirmary, England. Octavo of 379 pages, illustrated. Philadelphia and London: W. B. Saunders Company. 1910. (Cloth, $4.00; half morocco, $5.50 net prices.)

Contrary to the accepted belief of the recent past Mr. Moynihan asserts that duodenal ulcer is not only common, but its diagnosis is easy, or at least not very difficult, for the trained clinician to make. This is the first instance that a monograph has been published on this topic in which, indeed, Mr. Moynihan is a pioneer. Ulcers of the duodenum have a variety of causes. There are ulcers from burns or scalds, toxic in character no doubt, according to this author; uremic ulcers, associated with Bright's disease, dependent in some cases upon advanced change in the kidneys, in others there being no direct relationship with the renal lesion, which is coexistent with it; tuberculous ulcers, few in number, but operation has disclosed the lesions to be tuberculous in character; melena neonatorum associated with duodenal ulcer; these being the principal forms of this disease.

The symptoms are definite, not easily to be mistaken, and appear in an order and with a precision which are indeed remarkable, says Mr. Moynihan; and then he proceeds to give the characteristic symptoms of chronic duodenal ulcer. The treatment he says, too, always should be surgical. This is especially the case if there is perforation. The perforation of acute ulcer in typhoid fever permits of no alternative. But it is commonly the chronic ulcer that perforates.

In an appendix the cases that Mr. Moynihan had operated up to the end of 1908, one hundred and eighty-six in number, are published in detail. The record is prepared by Mr. Collinson, a colleague of the author. The mortality was 1.6 per cent. for the whole number, and among the last one hundred and twenty-one cases there was no death. This remarkable book will be received by the profession with approval, which should give special thanks to the author for placing before it this splendid monograph.

---

**The Sexual Life of Woman in its Physiological, Pathological and Hygienic Aspects.** By **E. Heinrich Kisch,** M.D., Professor of the German Faculty of the University of Prague. Translated by **M. Eden Paul,** M.D. Octavo, pp. 697. Illustrated. New York: Rebman Co. (Cloth, $5.00.)

Many attempts have been made of late to treat this delicate subject from the viewpoint of the scientific observer, and at the same time with sufficient clearness or plainness to meet the requirement of the class of readers who most need enlightenment on the topic dealt with in this book. Most of these writers have failed in one or both the purposes aimed at. Here, however, is a work that meets the requirements of the medical man as well as the woman who needs instruction on the essential points involved.

Kisch divides the sexual life of woman into three epochs: the onset of menstruation—the menarche; the culmination of sexual activity—the menacme; and the cessation of menstruation—the menopause. The first two are new, the third being the classic term to designate the cessation of menstruation. He discusses under these several divisions almost every feature of his subject, —anatomic, physiologic, and pathologic. He dwells with intelligent understanding and forceful reasoning upon these processes which are of far-reaching importance in their relation to the structure of society,—and considers them under such topics as the sexual impulse, copulation, conception and its prevention, fertility, sterility, determination of sex, and sexual hygiene.

The diseases incident to womankind are dealt with insofar as they may effect her sexual life, and particularly as they may serve to modify her sexual appetite or prevent fertility. Nowhere in the book is there an uninteresting paragraph, nor is there a chapter devoid of instructive features. It is a work capable of accomplishing much good, particularly if intelligently read and if its teachings are obeyed. Kisch says a young woman entering upon marriage should receive complete instructions beforehand regarding the sexual processes of copulation. How many mothers will obey the injunction? Kisch also says that the physician should warn the young husband, enlightening him as to these matters in order that the brutality of the nuptial night may be lessened and the defloration accomplished without undue violence, physical or mental. How many physicians will venture to give advice on this almost forbidden topic?

The book contains a number of illustrations, some of which could be improved, artistically speaking. But as we have already intimated, it is the best exposition of woman's sexual life we have seen, and, to be appreciated, it should be studied by every physician interested in the subject.

---

**Diseases of the Stomach and Intestines.** By **Robert Coleman Kemp,** M.D., professor of gastrointestinal diseases in the New York School of Clinical Medicine. Octavo, pp. 766. Illustrated. Philadelphia and London: W. B. Saunders Co. (Cloth, $6.00.)

The advances made in recent years in the diagnosis and treatment of diseases of the alimentary canal, have stimulated the production of much literature on the subject, not the least important of which is the work before us. This book is prepared with a view to aid the general practitioner in the selection of simple and practical methods. To demonstrate his way of handling his cases the author has appealed extensively to photography, thus conveying in many instances superb clinical pictures of his methods of diagnosis and treatment.

One of the pronounced, even special, features of this book, is the attention paid to visceral displacements. These questions are constantly assuming greater importance in the causation of disease, or at least in the symptomatology of abdominal affections. The mechanical treatment of visceral ptoses is described in much detail, and forms a most instructive feature of the book.

Another special feature of Kemp's treatise is his method of examination of the functions of the stomach. This and the local treatment of the stomach will prove of great value to the general physician. The chapter on diverticulitis is a novelty in a work of this kind, and should be studied with care. Many obscure symptoms will be explained through the information to be obtained in this chapter. The author includes typhoid fever in his treatise, very properly so, too, on account of its intestinal complications. Appendicitis, also, is accorded a place because, though a surgical disease, the physician of the family should make himself familiar with its earliest symptoms and should be able to make a differential diagnosis. This is a most instructive treatise and is entitled to occupy a central place in the library of the general practitioner, and a prominent one as well, among the selected works of the specialist. Kemp speaks well and clearly, and deserves the attentive ears of a large audience. The book is remarkably well indexed and the foot note references are voluminous.

---

**Diseases of the Eye.** By **G. E. deSchweinitz,** A.M., M.D., Professor of Ophthalmology in the University of Pennsylvania. Sixth edition. Octavo, pp. 945. Illustrated. Philadelphia and London: W. B. Saunders Co. 1910. (Cloth, $5.00.)

The library of no ophthalmologist would be complete without de Schweinitz's treatise, which he so modestly calls a manual. Indeed, could one possess but two or three works on the eye this one would be found among them, and perhaps the first one chosen. While its original purpose was to serve the beginner in the study of ophthalmology, it has become with each progressive edition more and more a necessity for the specialist. Several portions of this edition have been rewritten in whole or in part, while many sections or paragraphs are entirely new. It has been the aim of the author, who has reached the top rung of the ladder in this branch or department of medicine, to keep his book abreast of the progress constantly taking place in ophthalmological science. How well he has succeeded in accomplishing his purpose, can be ascertained by a critical examination of this book.

---

**Medical Electricity and Röntgen Rays.** By **Sinclair Tousey,** A.M., M.D., Consulting Surgeon to St. Bartholomew's Clinic, New York. Octavo of 1116 pages, with 750 illustrations, 16 in colors. Philadelphia and London: W. B. Saunders Company, 1910. (Cloth, $7.00; half morocco, $8.50, net prices.)

It is difficult to estimate the limitations or the worth of electricity as applicable to the treatment of disease. If one may judge, however, by the amount of literature on the subject that has appeared in recent years, it could be said with a truth, that there is almost no limit to its range, and that its worth is incalculable. Here is a work, for example, that contains more than a thousand pages of unleaded material, and nine hundred and fifty illustrations relating to the diagnosis and treatment of disease by electricity,—greater in size, indeed, than many earlier works on the theory and practice of medicine.

Tousey handles his work well; he does not claim too much for electricity but details facts that have been established by investigation. He first gives a few general considerations, and then proceeds to enumerate all sorts of instruments and appliances used in medical practice for the purpose of treating or diagnosticating disease.

The physiologic effects of electricity is the topic discussed in an interesting section of the book, in which many experiments are pointed out to sustain arguments, advanced regarding visceral and other structural effects of the currents. In the section on electrodiagnosis a number of plates are introduced to depict motor points on the surface of the several regions of the body. Ionic medication by electrolysis is described and applied in the treatment of several affections, chiefly neuroses or nevi. Static and dynamic electrotherapy form the topics of a section, electromagnets are described in short section and then comes electricity in diseases of the nervous system. This latter should prove one of the most attractive sections in the book to the electrotherapeutist. High-frequency currents are of special interest at this time and are well described and applied by Tousey. The transmission of electricity through gases produce interesting phenomena that are set forth in a section that details the processes involved; phototherapy is an interesting topic and is well described in a well illustrated section. But perhaps the sections that will be most appreciated are those pertaining to the *x*-ray, to Röntgentherapy, and to radium. Seldom have these topics been so adequately handled as by Tousey, and it is apparent that what he says will command attention. In short this entire treatise may be regarded as a distinct contribution to the literature of medical electricity.

---

**Surgical After-Treatment.** By **L. R. G. Crandon,** A.M., M.D. Assistant in Surgery at Harvard Medical School. Octavo of 803 pages, with 265 original illustrations. Philadelphia and London: W. B. Saunders Company, 1910. (Cloth, $6.00; half-morocco, $7.50 net prices.)

Not less important than a properly performed operation is well directed after-treatment, and we think Crandon has chosen well his material for the treatise or manual before us. Time was, and this, too, within the recollection of men now living, that the surgeon himself,—the operating surgeon, we mean,—attended to the after dressings of the wound, this being the case in the country in particular; though even in the city the surgeon generally supervised if, indeed, he did not in hospital actually change the dressings when occasion required.

Following so close upon Haubold's similar work which we dealt with in the July issue of this magazine, it would seem as if there were small need of further extended remark upon the subject. Nevertheless, though quite similar in scope, the two books differ in detail, hence deserve separate consideration as well as examination and study. Crandon well says: "the fact that each surgeon eventually grows into a technic peculiar to himself, and that many differing ways are successful, should make us liberal in spirit, and constantly alert for new truth."

This treatise, through its title would indicate its limitation to the after treatment of surgical patients, deals, nevertheless, with questions relating to the preparation of the patient for operation, as well as many other collateral topics germane to the general purpose of the book. The work is divided into two parts, the first being devoted to the initiative and concurrent topics and issues of the operation; and the second parts concerns itself, principally, with operations on special organs or in special regions. Beginning in the first part with the sick room it passes on to anesthesia, which it discusses with scientific acumen; then, almost every issue that may arise or follow on the trail of an operation is presented, until thirty-nine chapters, three hundred and fifty-seven pages, have been filled with material most instructive and practical in character. In this section almost every possible symptom or complication that can present itself is dealt with, and the technic of procedures to meet them are described. In the chapter relating to foreign bodies left in the abdomen, Crandon tells the story of a seal ring left in the abdominal cavity by one surgeon, removed later through the vagina by another operator, and finally, restored to the owner by the patient. We have heard this remarkable anecdote at first hands by one of the surgeons interested.

In the second part twelve chapters, about two hundred and forty-two pages, are taken up in considering operations in the various regions of the body, including those on the organs and tissues resultant from disease or injury. A chapter, number fifty-two, is devoted to therapeutic immunisation and vaccine therapy; one, number fifty-three, presents the use of the Coley serum for malignant tumors; an appendix follows giving some useful invalid and convalescent food recipes, and then comes comprehensive indexes of authors and subjects which close a valuable and instructive manual. It contains two hundred and sixty-five original illustrations, most of which are high class half-tone engravings. These are all well placed to elucidate or accentuate the text, but the number could be increased to advantage and no doubt will be in future editions. We congratulate the author in having added to the literature of surgery in such a satisfactory manner.

---

**Pulmonary Tuberculosis and Its Complications.** By **Sherman G. Bonney,** M.D., Professor of Medicine, Denver and Gross College of Medicine, Denver. Octavo of 955 pages, with 243 original illustrations. Philadelphia and London. W. B. Saunders Company. 1910. (Cloth, $7.00; half morocco, $8.50, net prices.)

Less than two years ago,—to be precise, in November, 1908,—we published a notice of the first edition of this treatise, and now comes the second edition for similar attention. Of the treatise when it first appeared we said it was essentially a clinical exposition of the subject, intended more particularly for the general practitioner and from this viewpoint possessed special value. We remarked, moreover, that the residence of the author within the zone of climatic prevention or cure, qualified him to speak with intelligence on this part of the subject,—a part, too, of no mean import. Since the isolation of the tubercle bacillus, views relating to the cause of pulmonary consumption have completely changed front, the doctrine of heredity even by many having been turned away as unworthy of credence.

The foregoing applies with equally cogent force to this new edition, and will bear the accentuation of repetition here. While it is impossible for any one author to cover the entire field of tuberculosis within the confines of a single book, yet it is surprising how many phases of the disease Bonney has presented, some of which he has dealt with elaborately. This volume contains nearly two hundred pages more than did the first edition, and when one reflects that it is a book of nine hundred and fifty-five pages, each of which is printed "solid," it can then be appreciated what an enormous amount of material has been crowded into its chapters.

It is proper to state that the author has made extremely careful revision of the entire text, rewriting much of it, and adding new matter which has appeared since the former edition was published, thus making the book as complete as such a treatise can be made. It represents and expounds the most modern thought relating to a disease that is second to none in the number of individuals it affects, or in the mortality it causes. Moreover, it is both preventable and curable, under the modern application of medical and sanitary science, and should be studied from the viewpoint that is paramount in this work—namely, that of the clinician.

---

**Modern Medicine.** Its Theory and Practice. Edited by **William Osler,** M.D., Regius Professor of Medicine in Oxford University, England. Assisted by **Thomas McCrea,** M.D., Associate Professor of Medicine and Clinical Therapeutics in Johns Hopkins University. Illustrated. Volume VII. Diseases of the Nervous System. Philadelphia and New York. 1909. Lea & Febiger, Publishers. (Per volume: cloth, $6.00; leather, $7.00; half morocco $7.50.)

The seventh and last volume of this remarkable work presents itself in the form of a comprehensive treatise on diseases of the nervous system, contributed by a distinguished group of neurologists. Of course, it is a surgeon, Harvey W. Cushing, who deals with tumors of the brain and meninges, and hydrocephalus. We have invited attention on another occasion to the fact that a neurologist, an ophthalmologist and a surgeon, should be associated in the diagnosis and treatment of brain neoplasms. We think it would have been well to have invited an ophthalmologist to write upon the eye symptoms of brain tumors for this work.

This volume will be sought by neurologists as being the latest exposition of the topic, each chapter being written by a man skilled in the science of nervous disease. Each subject therefore, becomes a monograph to which the author thereof has given his best thought. This makes the book authoritative, to be referred to by thousands of physicians in every quarter of the globe as the final judgment on any particular point upon which opinion is desired.

The whole treatise of seven volumes puts the owners of it in possession of a library containing near six thousand pages, appropriately illustrated wherever this is essential, and likewise well printed. The entire set has been prepared by the cooperation of editors, authors and publishers, under a skilfully devised plan, which has resulted in the creation of a complete library of present-day medicine within the convenient compass of about six thousand pages, adequately illustrated. Each volume is indexed, and the seventh contains a general index to the whole, so that the latest authoritative information on any point is immediately at command. For a work of such great value and usefulness a phenomenal demand was assured, hence was possible to fix a price low enough to meet the convenience of every practitioner who aims to obtain the best and latest literature for his bookshelves.

---

**Progressive Medicine**, Vol. 12, June, 1910. A Quarterly Digest of Advances, Discoveries and Improvements in the Medical and Surgical Sciences. Edited by **Hobart Amory Hare**, M.D., Professor of Therapeutics and Materia Medica in the Jefferson Medical College of Philadelphia and **Leighton F. Appleman**, instructor in therapeutics at the same college. Philadelphia and New York: Lea & Febiger. (Per annum, paper bound, $6.00; cloth, $9.00.)

The contributors to this number are Coley, hernia; Foote, surgery of the abdomen, except hernia; Clark, gynecology; Stengel, diseases of the blood, diathetic and metabolic diseases, diseases of the thyroid gland, nutrition, and the lymphatic system; and Jackson, ophthalmology. Coley's article comprises about fifty-five pages of well prepared material giving the latest features of the operation for hernia and sixteen excellent illustrations, most of which were executed by Miss Fry, one of the best of surgical artists. Foote presents near seventy-five pages pertaining to surgery of the abdomen replete with valuable substance. Ball's operation for pruritus ani, illustrated, being included in the record. Clark devotes some twenty-eight or thirty pages to cancer of the uterus, and about fifty pages more to miscellaneous topics. Most of his references are from the American Gynecological transactions. It might be well for the abstractor to inform himself of the fact that there are others. Stengel occupies over a hundred pages with his report on diseases of the blood, nutritional, and glandular affections. Jackson closes the book with twenty-five pages or more relating to ophthalmology; the whole making a valuable compilation of late literature pertaining to the several topics dealt with.

---

**New and Nonofficial Remedies.** Containing descriptions of articles which have been accepted by the Council in Pharmacy and Chemistry of the American Medical Association, prior to January 1, 1910. (Paper, 25 cents; cloth, 50 cents.)

This list is issued every year to indicate the work of the council on pharmacy and chemistry of the American Medical Association. Particular attention is invited to the descriptions of serums and vaccines, page 169. Since knowledge of the therapeutic value of new remedies is still largely in the experimental stage, the statements which appear under each proprietary article are based largely on the claims made by those interested. On the other hand, on page 56, under creosote carbonate, is a note on the claims of non-toxicity often made for certain remedies. A similar caution in reference to the claimed harmlessness of intestinal antiseptics appears on page 41 under beta-naphthol benzoate.

